# Neutralizing human monoclonal antibody against H5N1 influenza HA selected from a Fab-phage display library

**DOI:** 10.1186/1743-422X-5-130

**Published:** 2008-10-28

**Authors:** Angeline PC Lim, Conrad EZ Chan, Steven KK Wong, Annie HY Chan, Eng Eong Ooi, Brendon J Hanson

**Affiliations:** 1 Defence Medical and Environmental Research Institute, DSO National Laboratories, 27 Medical Dr, 117510, Singapore

## Abstract

Identification of neutralizing antibodies with specificity away from the traditional mutation prone antigenic regions, against the conserved regions of hemagglutinin from H5N1 influenza virus has the potential to provide a therapeutic option which can be developed ahead of time in preparation for a possible pandemic due to H5N1 viruses. In this study, we used a combination of panning strategies against the hemagglutinin (HA) of several antigenic distinct H5N1 isolates to bias selection of Fab-phage from a naïve human library away from the antigenic regions of HA, toward the more conserved portions of the protein. All of the identified Fab clones which showed binding to multiple antigenically distinct HA were converted to fully human IgG, and tested for their ability to neutralize the uptake of H5N1-virus like particles (VLP) into MDCK cells. Five of the antibodies which showed binding to the relatively conserved HA2 subunit of HA, exhibited neutralization of H5N1-VLP uptake in a dose dependant manner. The inhibitory effects of these five antibodies were similar to those observed with a previously described neutralizing antibody specific for the 140s antigenic loop present within HA1 and highlight the exciting possibility that these antibodies may be efficacious against multiple H5N1 strains.

## Background

Human disease due to direct transmission of highly pathogenic avian influenza A virus (HPAI) of the subtype H5N1 from poultry was first reported in 1997 and resulted in the death of 6 of the 18 infected individuals [1-3]. Re-emergence of HPAI-H5N1 viruses occurred in 2003 and to date has continued to be a cause of disease in both humans and poultry [4]. Currently H5N1 strains do not transmit efficiently between people, a trait that has probably limited the spread to the human population, and most human cases remain a result of a direct bird-to-human transmission [5] As at mid-January 2008, there have been 349 reported cases of human H5N1 infection with a high mortality rate resulting in the death of 216 individuals. Since 2003, increased geographical distribution (H5N1 has been reported in a variety of birds from over 50 countries) coupled with continued evolution of H5N1 viruses and an immunologically naïve human population has maintained the pandemic potential of these viruses [6,7].

The cornerstone of most pandemic preparedness plans is the stockpiling of antiviral drugs against the influenza virus. Two types of antiviral drugs are available for use against influenza, the M2 inhibitors and the neuraminidase inhibitors. However, the emergence of drug resistant influenza strains raises concern over their effectiveness. H5N1 viruses resistant to M2 inhibitors are widespread [8], and the development of resistance to the neuraminidase inhibitor, oseltamivir is emerging [9,10]. H5N1 strains exhibiting resistance to oseltamivir were initially thought to be less fit. However, recent studies have found that resistant viruses retain their replication efficiency and pathogenicity [11]. In addition, the effectiveness of the neuraminidase inhibitors appears to be very time dependant, where treatment started later than 24 hours post infection is much less effective [12]. Given this environment, mathematical modeling has predicted that should a pandemic H5N1 virus emerge with transmission characteristics similar to previous pandemic strains, containment strategies based solely on the use of antiviral drug therapy would be ineffective [13].

Recently, we and others have reported the therapeutic efficacy of passive immunization in a HPAI H5N1 mouse model with either humanized mouse mAb [14], equine F(ab')_2 _[15], or human mAb [16], directed against hemagglutinin (HA) of H5N1 influenza, highlighting its potential as a viable treatment option in human cases of H5N1. Indeed, survival of a person infected with HPAI H5N1 has been reported after treatment with convalescence plasma [17]. Influenza viruses rapidly mutate, particularly in the regions of HA responsible for antigenicity, and this has led to the emergence of multiple antigenically distinct strains of H5N1 [18], indicating that escape from the protective effects of neutralizing antibodies directed against the known antigenic regions may be rapid. For passive immunization to be useful as a defense against influenza pandemic, it will need to overcome such antigenic drifts.

We hypothesize that the development of therapeutic antibodies against epitopes that lie outside of the antigenic sites may provide some resistance against virus escape, and be more beneficial for use in passive immunotherapy. The ability to display antibody fragments on bacteriophage for selection allows strategies to be employed for isolation of specific antibodies not possible by the conventional animal immunization technologies [19]. This paper describes isolation of a number of Fab from a naïve human library, by sequential panning against HA from antigenically distinct H5N1 strains. The binding of the recombinant human antibodies to HA was shown to be independent of the common antigenic regions and several of the antibodies exhibited neutralization in a cell based neutralization assay using H5N1-VLP, highlighting their potential for use in passive immunization against H5N1 virus infection.

## Results

### Expression of Hemagglutinin

Focusing on mutations within the 140s and 150s antigenic loops, alignment of all the HA sequences from H5N1 viruses isolated throughout 2005 and 2006 deposited into the Influenza Virus Resource maintained by NCBI [20], revealed eight main groups. A representative of each of these groups was selected to be cloned for use in antibody screening and antibody binding analysis (Table 1).

**Table 1:** Position of mutation in HA1 compared to A/Vietnam/1203/04

Virus	Amino acid position in HA1								
	
	140s LOOP						150s LOOP		
	136	137	138	139	140	141	154	155	156
A/Vietnam/1203/04 (VN04)	P	Y	Q	G	K	S	N	S	T
A/Qing Hai/05 (QH)	**.**	**.**	**.**	**.**	R	**.**	**.**	N	A
A/WS/Mongolia/2/06 (MON)	**.**	**.**	**.**	**.**	R	**.**	**.**	D	A
A/DK/Vietnam/376/05 (VN05)	**.**	**.**	**.**	**.**	N	P	**.**	**.**	**.**
A/CK/Ivory Coast/1787/06 (IC)	**.**	H	**.**	**.**	R	**.**	D	N	A
A/Zhe Jiang/16/06 (ZHE)	**.**	**.**	**.**	**.**	T	P	**.**	N	**.**
A/Indonesia/CDC597/06 (IN597)	**.**	**.**	L	**.**	R	**.**	**.**	N	**.**
A/DK/Guangzhou/20/05 (GUAN)	**.**	**.**	L	**.**	**.**	P	**.**	N	A
A/Indonesia/5/05 (INDO05)	**.**	**.**	L	**.**	S	P	**.**	**.**	**.**
A/CK/Indonesia/R60/05 (CKINDO)	S	**.**	L	**.**	D	P	**.**	**.**	A

^*a *^residues similar to A/Vietnam/1203/04 are marked by a period

**Figure 1:**
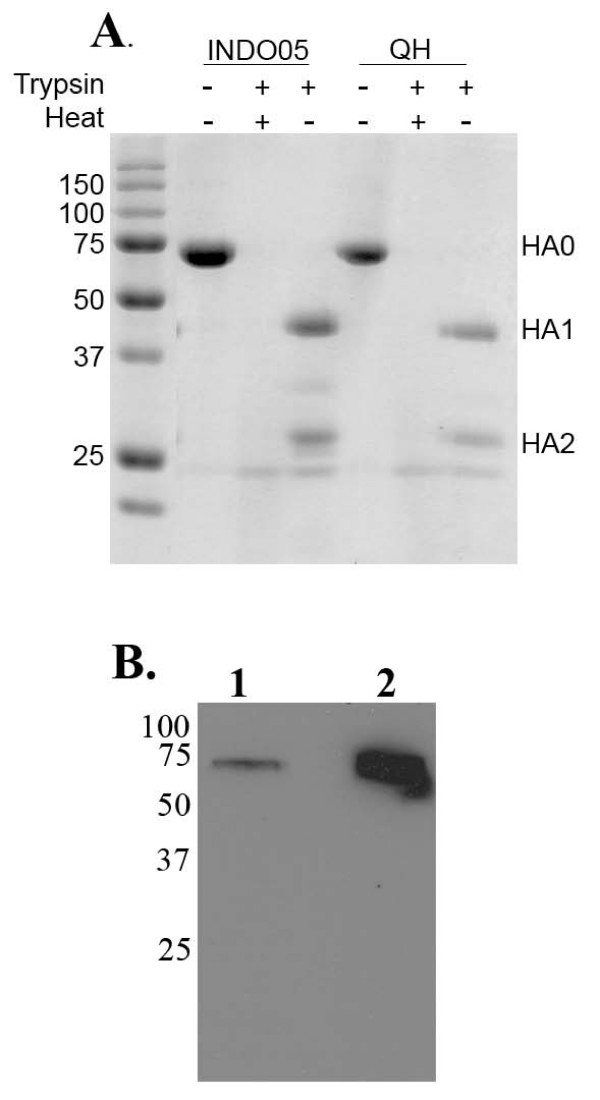
**Analysis of HA-FO and H5-VLP**. (A) The indicated HA foldons from recombinant baculovirus were collected and allowed to incubate with Talon resin. Following elution, equal amounts were treated with trypsin to check for correct folding, heat denaturation was used as a control for trypsin activity. Proteins were resolved on 12% SDS-PAGE and stained with coomassie. Mw markers (sizes in kDa, left) and the bands representing HA0, HA1 and HA2 are indicated. (B) H5-VLP were released from HEK293 cells by addition of exogenous NA, the VLP pellet after sucrose cushion purification was resolved on 12% SDS-PAGE. Immunoblots were probed with α-HA (VN04-2).

Full-length HAs were constructed by replacing the transmembrane domain present in the HA2 subunit with the trimerization domain 'foldon' of bacteriophage T4 fibritin (FO), allowing secretion of functionally folded HA trimers [21]. The HA2 subunit of A/Vietnam/1203/04 HA was mutated to contain the FO sequence and ligated to the individual HA1 described in table 1 and recombinant baculoviruses were produced by infection of Sf9 cells with the respective pENTR-HA-FO. The cell medium was collected 60 hours after infection of Sf9 cells with the recombinant baculovirus and allowed to incubate with hexa-histidine affinity beads (Talon) for two hours at room temperature. Bound proteins were eluted with 150 mM Imidazole, subjected to trypsin digestion to check for correct folding and resolved on 12%SDS-PAGE (Fig. 1A). The presence of a ~75 kDa protein which was cleaved into an ~40 kDa and ~27 kDa with trypsin suggested the purified protein was indeed correctly folded HA. This protein was used for screening against the Fab-library.

For library panning purposes, H5-VLPs were produced by the transient expression of MMLV retroviral core and H5 HA alone to HEK293. This was to prevent the selection of antibodies from the Fab library also binding to NA. It has been shown that HA was incorporated on the surface of the viral particles even without the presence of endogenous NA or M2, and that these could be released with the addition of exogenous neuraminidase [22]. The production and release of VN04 H5-VLP using exogenous NA was determined by looking for the presence of HA by immunoblotting using our humanized anti-HA antibody VN04-2 [14]. Significant levels of released VN04 H5-VLP purified on a 30% sucrose cushion, could only be detected with the addition of exogenous NA (Fig. 1B).

### Isolation of anti-H5 Fab-phage

Two different approaches of screening the Humanyx Fab library were devised to bias selection away from the antigenic regions. In the first approach, panning was performed completely against recombinant baculovirus encoded HAs expressed in insect cells. Phage displayed Fabs were first selected several times on immunotubes coated with VN04-PS, followed by QH-FO in order to increase the binding diversity of the Fabs. After the final pan against QH-FO, the Fab from 380 single colonies were tested for their ability to bind to VN04-PS by ELISA, BstN1 digest of the 172 positive clones allowed grouping of clones with similar patterns. Sequencing confirmed the identity of 18 unique clones with the ability to bind to all of the HA-FO described in table 1 in a confirmatory monoclonal ELISA (Table 2).

**Table 2:** Selection of phage displayed Fabs

H5 Target	Total	Positive	Unique	Clone
	pans	clones	clones	identity
VN04-PS/QH-FO	4/2	172	18	HA1-18
INDO05-FO/VN04-VLP/QH-VLP	2/1/1	25	15	VLP1-15

In the second approach, panning was performed against a combination of recombinant baculovirus encoded HA and H5-virus like particles (VLPs). INDO05-FO was bound onto magnetic Nickel-coated affinity sepharose through its hexa-histidine tag on the HA2, of which the library was put through the first two rounds of selection. The amplified Fab phages were further selected on immunotubes coated with QH-VLP, followed by final selection on VN04-VLP. Placing the initial selection pressure on a HA-FO protein ensured that the selection of Fabs was not directed towards epitopes on the VLPs. Analysis of the Fab from 190 single colonies identified 25 positives by ELISA against INDO05-FO, all of which were sequenced to reveal 15 Fab clones with distinct immunoglobulin sequences that bound to VN04- and QH-VLPs, as well as INDO05-FO and QH-FO in a confirmatory monoclonal ELISA (Table 2).

All 33 Fabs were subsequently converted into IgGs and tested for their binding specificities and neutralizing abilities. Purity of the IgGs was verified using SDS-PAGE analysis (data not shown) and all but one (αHA2) of the IgGs could be expressed and purified.

### Subunit specificity of Fab derived human IgGs

**Figure 2:**
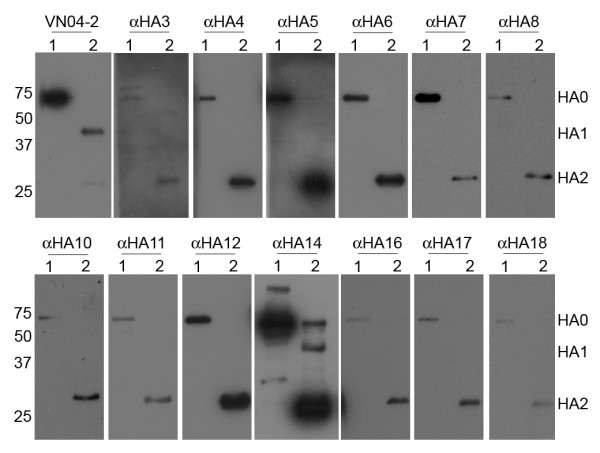
**HA subunit specificity of Fab derived human IgGs**. Purified IN597-FO was resolved on reducing SDS-PAGE and transferred to Immunoblots probed with anti-HA Fab-IgGs. Goat anti-human (peroxidase conjugated) was used as the secondary signal producing antibody. 1-Untreated IN597-FO; 2-Trypsin-cleaved IN597-FO. The humanized protective antibody VN04-2 was used as a positive control. for presence of proteins on blot.

To determine the domain that our IgGs bind to, HA of the A/Indonesia/CDC597/06 (IN597-FO) strain was subjected to trypsin digestion and resolved into the HA1 and HA2 subunits by reducing SDS-PAGE, followed by immunobloting with the IgGs. The majority of the antibodies that were obtained from the VN04-PS/QH-FO screen appeared to recognize linear epitopes as they could detect reduced antigen; except for αHA1 which showed no binding and αHA14 which seemed to bind to both HA1 and HA2, all of the antibodies bound to HA2 (Fig. 2) In contrast, the antibodies obtained from the INDO05-FO/VN04-VLP/QH-VLP screen appeared to recognize conformational epitopes, as none of the antibodies bound to reduced HA on the immunoblots (data not shown).

### Inhibition of H5N1-VLP uptake in MDCK cells by αHA antibodies

**Figure 3:**
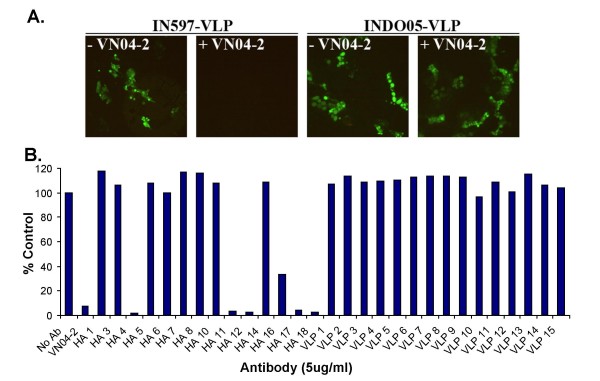
**H5N1-VLP neutralization assays with Fab derived human IgGs**. H5N1-VLP were produced in HEK293 cells and the media incubated with 5 ug/ml of the indicated IgG for 60 min prior to addition to MDCK cells. (A) Initial validation of the VLP neutralization assay was performed using IN597- and INDO05-VLP with and without VN04-2, fusion of the VLP with MDCK was visualized by immunofluorescence microscopy. (B) Testing of the isolated IgG was performed against IN597-VLP and fusion of the VLP with MDCK was determined by measuring the number of cells producing GFP by flow cytometry with the control assay with no IgG taken as 100%. The humanized protective antibody VN04-2 was used as a positive control.

Recently, retroviral core particle derived virus-like particles (VLP) harboring the surface proteins of Venezuelan equine encephalitis virus and H5N1 have shown their potential as vaccine candidates and also through inclusion of either luciferase or GFP reporter genes, utility as a substitute for live virus in cell based neutralization assays [22-24]. When developing the VLP neutralizing assay, to ensure that uptake of H5N1-VLP into MDCK cells was similar to the genuine virus infection, we initially used H5s where the polybasic cleavage site at the end of HA1 had been replaced with a trypsin cleavage site: addition of these VLPs to MDCK in the absence of trypsin did not allow visualization of GFP by immunofluoresence microscopy suggesting that the VLP was unable to fuse with the cells (data not shown). When the same experiment was repeated using H5 containing the polybasic cleavage site, GFP could be observed (Fig. 3A). In addition, our previously described protective antibody VN04-2 could inhibit uptake of IN597- but not INDO05-VLP (Fig. 3A), the latter of which does not bind the VN04-2 antibody [23]. The necessity of HA cleavage for GFP reporter gene integration and the inhibitory characteristics displayed by VN04-2, suggests that H5N1-VLP enter MDCK cells in a similar mechanism to genuine H5N1 virus.

To test the neutralization potential of our antibodies, all 32 antibodies were tested for their neutralizing efficacy using IN597-VLP containing the GFP reporter gene. Each antibody was allowed to incubate with IN597-VLP for 60 min prior to addition to MDCK cells and the number of cells infected with the VLP was determined after 72 hr, 5 of the antibodies (αHA IgGs #4, #11, #12, #17, #18) were able to neutralize IN597-VLP infection (Fig. 3B).

**Figure 4:**
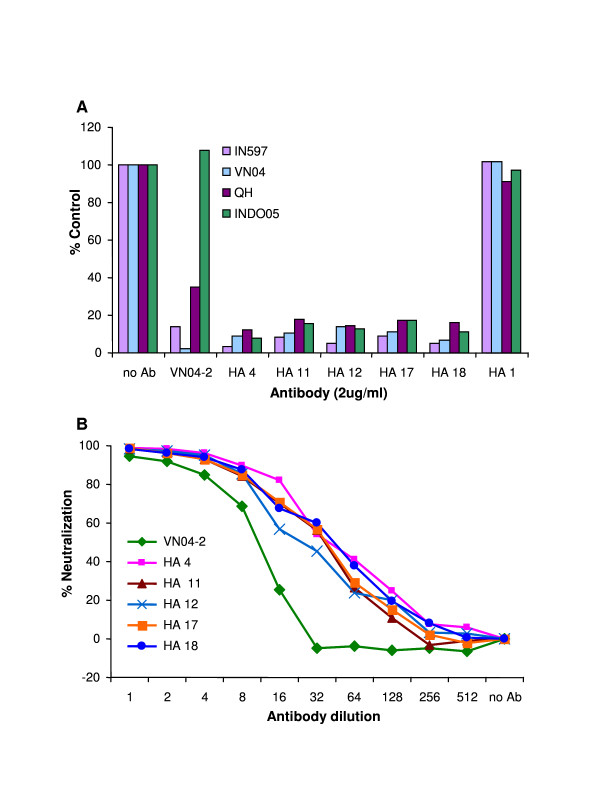
**H5N1-VLP neutralization characteristics of αHA Fab IgG**. (A) To confirm the neutralization observed with the indicated α-HA Fab IgG, neutralization assays were repeated. IN597 and VN04 were produced with the HA2 of VN04 while QH and INDO05 were produced with their respective HA2 in HEK293 cells and the media incubated with 2 ug/ml of the indicated IgG for 60 min prior to addition to MDCK cells. Fusion of the VLP with MDCK was determined by measuring the number of cells producing GFP by flow cytometry with the control assay with no IgG taken as 100%. (B) Dose dependant neutralization was assessed against IN597 H5N1-VLPs, with 8 ug/ml and serial dilutions of the indicated antibodies, as described above. The humanized protective antibody VN04-2 was used as a positive control.

To confirm this set of data, the concentration of antibodies used in the neutralization assay was lowered from 5 ug/ml to 2 ug/ml. At the same time, neutralization against VN04, INDO05 and QH VLPs were also tested as these H5-VLPs contain complete HA0 sequences that correspond fully to their genuine virus. While the HA2 region of H5 does show some genetic variability, the N-terminal portion of the subunit, containing the fusion peptide is highly conserved. Indeed, for the H5s used here mutation is only present in the c-terminal third of HA2. With 2 ug/ml of IgGs (αHA IgGs #4, #11, #12, #17, #18) used in the assay, above 90% neutralization was still observed with IN597 and above 80% with VN04-, INDO05- and QH-VLP (Fig. 4A). To investigate whether the neutralization of H5N1-VLPs was similar to that observed with a known protective antibody, the antibodies were titrated and the effects on inhibition of H5N1-VLP uptake were observed and compared to that of the neutralizing antibody VN04-2 [14]. All 5 of the antibodies showed a dose dependant response which showed similar characteristics to VN04-2 (Fig. 4B).

## Discussion

By using a combination of screening strategies which involved sequential panning against antigenically distinct HA of H5N1, we were able to isolate from a naïve human Fab-phage library 33 unique Fab which exhibited binding to multiple distinct HA representative of the major antigenic changes which had occurred throughout 2005 and 2006. In a cell based neutralization assay using H5N1-VLP, 5 of these antibodies which show specificity for HA2 were found to be neutralizing in a dose dependant manner, similar to our previously described protective humanized antibody, VN04-2, which is against the 140s antigenic loop present in the HA1 subunit [14].

The majority of studies describing neutralizing antibodies against influenza HA has utilized either H1N1 or H3N2 and has lead to the characterization of the antigenic regions within HA1 and underlined inhibition of virus-cell interaction as the main mechanism [25,26]. Determination of the structure of H5 has highlighted the similarity in the protein fold to HA of other subtypes [21] and studies using H5 have shown that neutralizing antibodies bind to similar antigenic regions [14,16,27,28]. Indeed, neutralizing antibodies have been used to determine the extent of antigenic variation within H5N1 isolates [18] and comparison of the amino acid sequence of H5N1 isolates shows extensive mutation within these regions, affirming their designation as major antigenic regions. Variations in the HA antigenic regions are created as passage of virus through the animal/human hosts produces selective pressure which favours mutation within these regions. As such areas of the protein outside of these regions are relatively conserved, increasing the likelihood that these regions will be unchanged in a future pandemic strain of H5N1.

In contrast to HA1, HA2 which is primarily responsible for mediating the fusion of viral and cell membranes is relatively conserved. While this may suggest that HA2 is non immunogenic, natural infection with influenza as well as vaccination does induce antibodies against HA2 [29,30]. Studies of antibodies against HA2 have highlighted their high crossreactivity [31]. However, except for an antibody against a conformational epitope formed by HA1 and HA2 which inhibits conformational changes necessary for fusion [32], these antibodies have been found to be non-neutralizing *in vitro *[33,34], even though some of these antibodies are capable of inhibiting cell-cell and virus-liposome fusion assays [35]. Recently, despite the lack of neutralization *in vitro*, passive administration of antibodies against HA2, particularly those which can inhibit the fusion activity of HA, have been shown to be protective in a H3N2 influenza mouse model [36]. The five antibodies described here may be the first *in vitro *neutralizing antibodies specific for HA2, raising the exciting possibility that these antibodies may be protective against H5N1 influenza infection *in vivo*. However, it also raises the question as to why these antibodies have not been raised before, when antibodies specific for HA2 are usually non-neutralizing.

One possibility is that the combination of in vitro selection of Fab-phage and sequential panning against different HA variants may have allowed for the identification of antibodies against epitopes that are not usually open to antibody selection during the immune response against HA, be it from natural infection or vaccination. However, it also possible that the correlation between neutralization activity against VLP and virus observed for antibodies against Venezuelan equine encephalitis virus may not apply for these antibodies against H5 hemagglutinin. Further study of these antibodies is needed to determine their actual *in vivo *protective capacity. Examination of the in vivo efficacy of these antibodies as well as the mechanism of neutralization will be the focus of further investigation.

## Methods

### H5 Hemagglutinin HA1

The HA strains were chosen based on the differences to A/Vietnam/1203/04 (H5N1) in the main antigenic regions as shown in Table 1 and their cloning has been described previously [23]. Briefly, the cDNAs encoding the HA1 subunits of the selected HAs listed in Table 1 were produced by a combination of PCR-based methods and the polybasic protease site (RRRKKR) at the end of HA1 was replaced by the sequence PQIETR, which allows cleavage of HA0 by trypsin. The fidelity of each clone was confirmed by sequencing.

### Baculovirus expression

In order to produce soluble HA proteins, we used the recombinant baculovirus expression method for determination of the H5 HA structure [21]. Briefly, the transmembrane domain present in HA2 subunit of VN04 HA had been replaced by the 'foldon' (FO) trimerization sequence, followed by a hexa His-tag at the extreme C-terminus of the construct to enable protein purification. Full-length HA-FOs were cloned into the pENTR vector by combining the selected HA1 together with the VN04 HA2-FO and recombinant baculovirus were produced using the BaculoDirectTM Baculovirus expression system (Invitrogen). For protein expression, the recombinant baculoviruses were used to infect attached Sf9 cells. Sixty hours after infection, recombinant HA proteins secreted into the cell culture media were purified using talon affinity resin (Clontech). Purified HA of A/Vietnam/1203/04 produced in baculovirus (VN04-PS) was purchased from Protein Sciences Corp.

### H5N1 Virus-like Particles (VLPs)

H5N1-VLPs were produced using non-replicating viral core particles of the Moloney Murine Leukaemia Virus (MMLV) and the surface proteins of H5N1 as described previously [23]. Plasmids encoding the non-replicating MMLV core particle and GFP reporter gene, pVPack-GP and pFB-hrGFP respectively were purchased from Stratagene. For expression of H5 HA, the HA1 cDNAs (listed in table 1), together with HA2 were cloned into the CMV promoter driven expression vector, pXJ. For VN04, QH and INDO05, their respective HA2 was used; for IN597, the HA2 of VN04 was used. Following construction of the full length HA, the polybasic cleavage site at the end of HA1 was restored and all constructs were confirmed by sequencing. For expression of N1, the N1 neuraminidase (NA) of A/Vietnam/1203/04 was synthesized (GeneScript) and then cloned into pCI vector (Promega).

To produce H5-VLP for library panning, plasmids encoding the core particle and H5 were transiently transfected into HEK293 according to the protocol provided with the MBS Mammalian Transfection Kit (Stratagene). Thirty-six hours after transfection purified Vibrio Cholera neuraminidase (Roche) was added at 2 U/ml, VLPs were collected forty-eight hours post-transfection by ultracentrifugation through a 20% sucrose gradient at 22,000 rpm at 4°C for 2 hours.

To produce VLP for neutralization assays, plasmids encoding the core particle, H5, VN04-N1 and the GFP reporter were transfected as above, forty-eight hours following transfection, the culture medium was collected to be used in the assay.

### Phage display Fab library screening

Library screening was performed using the naïve human Fab phage display library HX01 (Humanyx Pte Ltd, Singapore). Antigen targets (HA or H5-VLP) were coated onto Nunc brand Maxisorb Immunotube or affinity magnetic beads (where indicated) at 4°C overnight. The coated tubes/beads and the phage library were separately blocked in 2% skim milk (Sigma) in PBS for 1 h at room temperature (RT). Pre-blocked phage mixtures were then incubated with the coated tube/beads for 2 h at RT, unbound phages were eliminated by washing 3 times with 2% skim milk in PBS-T(0.05% Tween 20), 3 times with PBS-T and twice with PBS. For subsequent pans, the washings were increased to 7 times with 2% skim milk in PBS-T, 7 times with PBS-T and twice with PBS. Bound phages were eluted either with 0.5 M triethylamine (TEA) for 10 min at RT or 2 mg/ml trypsin solution for 30 min at 37°C. Eluted phages were used to infect Escherichia coli TG1 cells grown to OD600 0.5 and subsequently rescued with M13-K07 helper phage. The rescued phages were amplified and plated on 2xTY-agar plates containing 100 ug/ml ampicillin and 25 ug/ml kanamycin and incubated at 30°C overnight. Plates containing bacteria were scraped into TBS and purified by PEG precipitation, the same procedure was repeated for subsequent pans, usually up to 6 pans were performed. To select for individual antigen-binding Fab clones, phage-infected TG1 cultures were titrated on 2xTY-agar plates containing 100 ug/ml ampicillin and 2% glucose. Single colonies were picked the next day into 96-well plates for expression of Fabs and tested for antigen recognition properties.

### Selection of monoclonal Fab

For expression of Fabs, cultures at ~OD600 0.5 were induced with 1 mM IPTG and incubated with shaking at 30°C overnight in 96-well plates. Screening of HA-binding Fabs was performed according to standard ELISA method. Maxisorb 96-well plates (Nunc) were coated with 2 ug/ml HA-FO per well at 4°C overnight and blocked with 4% skim milk in PBS at RT for 1–2 h. Culture supernatants, diluted 1:1 in blocking buffer, were added to the coated plates and allowed to incubate for 90 min at RT. Antigen recognitions were detected by peroxidase-conjugated anti-cMyc secondary antibody, followed by the addition of 3.3', 5.5'-tetramethylbenzidine substrate (Pierce). Clones producing absorbance values 2-fold higher than background levels were considered to be positive.

To assess the uniqueness of positive clones, BstN1 restriction digest was performed following PCR amplification of the Fab coding region and resolved on 3% Agarose gel. Clones showing similar patterns were grouped and the identity of the clones was determined by sequencing using specific primers annealing to the regions of the vector flanking the variable heavy and variable light chains of the antibody fragment, following plasmid extraction using Miniprep kits (QIAGEN).

### Conversion of Fab-phage to human IgG1

Distinct immunoglobulin sequences were converted to IgG1 by cloning the Fab our Fab-IgG1 vector through Apa L1 and Bsm BI. The Fab-IgG1 vector was adapted from the vector used to produce chimeric VN04-2 previously [14]. Changes to the cloning sites were performed to promote ease of transferring the variable domains from the Fabs directly to the IgG1 vector, without affecting the constant domains within the vector. Constructs encoding the Fab-IgGs were transfected into human embryonic kidney (HEK293) cells by use of lipofectamine 2000 (Invitrogen). Culture media was collected 72 h post transfection and fresh media was added to the cells and allowed to incubate for a further 72 h before collection. Secreted antibodies were purified using recombinant Protein A sepharose (Pierce). Purity of the Fab-IgGs was verified using SDS-PAGE analysis.

### H5N1-VLP neutralization assay

All the HA0 constructs used to produce H5N1-VLP that were used for neutralization assays contained the polybasic protease site between HA1 and HA2. H5N1-VLP containing the surface proteins H5 HA, VN04 NA and GFP reporter gene were produced by transient transfection as described above. At 48 h post-transfection, the viral supernatant were collected and filtered through a 0.45 micron filter. The filtered supernatant was then diluted 1:3 in growth medium and incubated with the test antibody at RT for 1 h. DEAE-dextran solution was added to the VLP-antibody mix to a final concentration of 10 ug/ml, before transferring the solution to Madin-Darby Canine Kidney (MDCK) cells and incubated at 37°C with 5% CO2. The cell media was replaced with fresh growth medium after 3 h. Transduction titre was deduced 72 h later from the number of GFP-positive cells measured by flow cytometry (FACSCalibur; Beckman Coulter). For experiments using more than one H5N1-VLP, HA units of each H5N1 was determined using the standard hemagglutination assay with 0.5% chicken red blood cells and the input of each H5N1-VLP standardized.

## Competing interests

The authors declare that they have no competing interests

## Authors' contributions

APCL, OEE and BJH conceived the study. APCL and BJH planned the experimental design, performed the baculovirus and VLP work and drafted the manuscript. CEZC helped with the Fab-phage library screening. SKKW participated in the design and performance of HA1 cloning strategies. AHYC helped with HA1 cloning and provided general technical assistance. All authors critically reviewed and approved the final manuscript.

## References

[B1] Claas EC, Osterhaus AD, van Beek R, De Jong JC, Rimmelzwaan GF, Senne DA, Krauss S, Shortridge KF, Webster RG (1998). Human influenza A H5N1 virus related to a highly pathogenic avian influenza virus. Lancet.

[B2] de Jong JC, Claas EC, Osterhaus AD, Webster RG, Lim WL (1997). A pandemic warning?. Nature.

[B3] Yuen KY, Chan PK, Peiris M, Tsang DN, Que TL, Shortridge KF, Cheung PT, To WK, Ho ET, Sung R, Cheng AF (1998). Clinical features and rapid viral diagnosis of human disease associated with avian influenza A H5N1 virus. Lancet.

[B4] Gillim-Ross L, Subbarao K (2006). Emerging respiratory viruses: challenges and vaccine strategies. Clin Microbiol Rev.

[B5] Ungchusak K, Auewarakul P, Dowell SF, Kitphati R, Auwanit W, Puthavathana P, Uiprasertkul M, Boonnak K, Pittayawonganon C, Cox NJ (2005). Probable person-to-person transmission of avian influenza A (H5N1). N Engl J Med.

[B6] Fouchier R, Kuiken T, Rimmelzwaan G, Osterhaus A (2005). Global task force for influenza. Nature.

[B7] Webster RG, Govorkova EA (2006). H5N1 influenza–continuing evolution and spread. N Engl J Med.

[B8] Li KS, Guan Y, Wang J, Smith GJ, Xu KM, Duan L, Rahardjo AP, Puthavathana P, Buranathai C, Nguyen TD (2004). Genesis of a highly pathogenic and potentially pandemic H5N1 influenza virus in eastern Asia. Nature.

[B9] Le QM, Kiso M, Someya K, Sakai YT, Nguyen TH, Nguyen KHL, Pham ND, Ngyen HH, Yamada S, Muramoto Y (2005). Avian flu: Isolation of drug-resistant H5N1 virus. Nature.

[B10] de Jong MD, Thanh TT, Khanh TH, Hien VM, Smith GJD, Chau NV, Cam BV, Qui PT, Ha DQ, Guan Y (2005). Oseltamivir Resistance during Treatment of Influenza A (H5N1) Infection. N Engl J Med.

[B11] Chen YS, Hsiao YS, Lin HH, Yen CM, Chen SC, Chen YL (2006). Immunogenicity and anti-Burkholderia pseudomallei activity in Balb/c mice immunized with plasmid DNA encoding flagellin. Vaccine.

[B12] Govorkova EA, Ilyushina NA, Boltz DA, Douglas A, Yilmaz N, Webster RG (2007). Efficacy of oseltamivir therapy in ferrets inoculated with different clades of H5N1 influenza virus. Antimicrob Agents Chemother.

[B13] Alexander ME, Bowman CS, Feng Z, Gardam M, Moghadas SM, Rost G, Wu J, Yan P (2007). Emergence of drug resistance: implications for antiviral control of pandemic influenza. Proc Biol Sci.

[B14] Hanson BJ, Boon AC, Lim AP, Webb A, Ooi EE, Webby RJ (2006). Passive immunoprophylaxis and therapy with humanized monoclonal antibody specific for influenza A H5 hemagglutinin in mice. Respir Res.

[B15] Lu J, Guo Z, Pan X, Wang G, Zhang D, Li Y, Tan B, Ouyang L, Yu X (2006). Passive immunotherapy for influenza A H5N1 virus infection with equine hyperimmune globulin F(ab')2 in mice. Respiratory Research.

[B16] Simmons CP, Bernasconi NL, Suguitan AL, Mills K, Ward JM, Chau NV, Hien TT, Sallusto F, Ha do Q, Farrar J (2007). Prophylactic and therapeutic efficacy of human monoclonal antibodies against H5N1 influenza. PLoS Med.

[B17] Zhou B, Zhong N, Guan Y (2007). Treatment with convalescent plasma for influenza A (H5N1) infection. N Engl J Med.

[B18] Chen H, Smith GJ, Li KS, Wang J, Fan XH, Rayner JM, Vijaykrishna D, Zhang JX, Zhang LJ, Guo CT (2006). Establishment of multiple sublineages of H5N1 influenza virus in Asia: Implications for pandemic control. Proc Natl Acad Sci USA.

[B19] Winter G, Griffiths AD, Hawkins RE, Hoogenboom HR (1994). Making antibodies by phage display technology. Annu Rev Immunol.

[B20] Bao Y, Bolotov P, Dernovoy D, Kiryutin B, Zaslavsky L, Tatusova T, Ostell J, Lipman D (2008). The Influenza Virus Resource at the National Center for Biotechnology Information. J Virol.

[B21] Stevens J, Blixt O, Tumpey TM, Taubenberger JK, Paulson JC, Wilson IA (2006). Structure and receptor specificity of the hemagglutinin from an H5N1 influenza virus. Science.

[B22] Szecsi J, Boson B, Johnsson P, Dupeyrot-Lacas P, Matrosovich M, Klenk HD, Klatzmann D, Volchkov V, Cosset FL (2006). Induction of neutralising antibodies by virus-like particles harbouring surface proteins from highly pathogenic H5N1 and H7N1 influenza viruses. Virol J.

[B23] Lim AP, Wong SK, Chan AH, Chan CE, Ooi EE, Hanson BJ (2008). Epitope characterization of the protective monoclonal antibody VN04-2 shows broadly neutralizing activity against highly pathogenic H5N1. Virol J.

[B24] Kolokoltsov AA, Wang E, Colpitts TM, Weaver SC, Davey RA (2006). Pseudotyped viruses permit rapid detection of neutralizing antibodies in human and equine serum against Venezuelan equine encephalitis virus. Am J Trop Med Hyg.

[B25] Knossow M, Gaudier M, Douglas A, Barrere B, Bizebard T, Barbey C, Gigant B, Skehel JJ (2002). Mechanism of neutralization of influenza virus infectivity by antibodies. Virology.

[B26] Skehel JJ, Wiley DC (2000). Receptor binding and membrane fusion in virus entry: the influenza hemagglutinin. Annu Rev Biochem.

[B27] Kaverin NV, Rudneva IA, Govorkova EA, Timofeeva TA, Shilov AA, Kochergin-Nikitsky KS, Krylov PS, Webster RG (2007). Epitope mapping of the hemagglutinin molecule of a highly pathogenic H5N1 influenza virus by using monoclonal antibodies. J Virol.

[B28] Kaverin NV, Rudneva IA, Ilyushina NA, Varich NL, Lipatov AS, Smirnov YA, Govorkova EA, Gitelman AK, Lvov DK, Webster RG (2002). Structure of antigenic sites on the haemagglutinin molecule of H5 avian influenza virus and phenotypic variation of escape mutants. J Gen Virol.

[B29] Cox RJ, Brokstad KA (1999). The postvaccination antibody response to influenza virus proteins. Apmis.

[B30] Styk B, Russ G, Polakova K (1979). Antigenic glycopolypeptides HA1 and HA2 of influenza virus haemagglutinin. IV. Immunogenic properties of separated haemagglutinin glycopolypeptides. Acta Virol.

[B31] Graves PN, Schulman JL, Young JF, Palese P (1983). Preparation of influenza virus subviral particles lacking the HA1 subunit of hemagglutinin: unmasking of cross-reactive HA2 determinants. Virology.

[B32] Okuno Y, Isegawa Y, Sasao F, Ueda S (1993). A common neutralizing epitope conserved between the hemagglutinins of influenza A virus H1 and H2 strains. J Virol.

[B33] Russ G, Polakova K, Kostolansky F, Styk B, Vancikova M (1987). Monoclonal antibodies to glycopolypeptides HA1 and HA2 of influenza virus haemagglutinin. Acta Virol.

[B34] Sanchez-Fauquier A, Villanueva N, Melero JA (1987). Isolation of cross-reactive, subtype-specific monoclonal antibodies against influenza virus HA1 and HA2 hemagglutinin subunits. Arch Virol.

[B35] Vareckova E, Mucha V, Wharton SA, Kostolansky F (2003). Inhibition of fusion activity of influenza A haemagglutinin mediated by HA2-specific monoclonal antibodies. Arch Virol.

[B36] Gocnik M, Fislova T, Sladkova T, Mucha V, Kostolansky F, Vareckova E (2007). Antibodies specific to the HA2 glycopolypeptide of influenza A virus haemagglutinin with fusion-inhibition activity contribute to the protection of mice against lethal infection. J Gen Virol.

